# Type D Personality and Big Five Personality Traits and the Risk of Breast Cancer: A Case-Control Study

**DOI:** 10.3389/fpsyt.2022.723795

**Published:** 2022-02-22

**Authors:** Irena Wojciechowska, Rafał Matkowski, Tomasz Pawłowski

**Affiliations:** ^1^Division of Psychotherapy and Psychosomatic Medicine, Wroclaw Medical University, Wroclaw, Poland; ^2^Wroclaw Comprehensive Cancer Center, Wroclaw, Poland; ^3^Department of Oncology, Wroclaw Medical University, Wroclaw, Poland

**Keywords:** breast cancer, Type D personality, big five personality, case-control, NEO–five-factor inventory (NEO-FFI)

## Abstract

**Objective:**

The goal of this study is to establish the differences in Type D personality and Big five personality traits between a group of newly diagnosed breast cancer patients and a group of controls.

**Methods:**

A comparative study of breast cancer patients and women without previous history of cancer was carried out. We used Type D Scale-14 as an instrument for the assessment of the type-D personality pattern and NEO-FFI for the assessment of the Big Five personality traits. Conditional logistic regression models were used to estimate odds ratios and 95% confidence intervals were applied for breast cancer by personality trait factors.

**Results:**

Negative affectivity (NA) (OR = 4.45 95% CI: 1.96–10.61), neuroticism HIGH (OR = 3.97, 95% CI: 1.08–15.81), openness to experience HIGH (OR = 3.47 95% CI: 1.11–11.49), were associated factors significantly related to an increased risk of breast cancer, whereas Social Inhibition (SI) was associated factor with a decreased risk of breast cancer (OR = 0.40 95% CI: 0.16–0.92).

**Conclusions:**

This was the first case-control study which analyzed NA and SI traits in breast cancer patients. SI as a breast-cancer risk decreasing factor might indicate that expressing negative emotions is not always a healthy mechanism of their regulation.

## Background

The relationship between cancer and personality causes strong controversy, indirectly leading to posing the question of whether cancer is a psychosomatic disease. The research of Greer and Morris on a group of women with breast cancer defined the behavior pattern later named Type C behavior (1, 2), also referred to as Type C (cancer-prone) personality (3). Type C personality is associated with neuroticism and introversion. The research of Rymarczyk et al. proposed a two-facet structure of Type C which is composed of submissiveness and restricted affectivity (4). Submissiveness (the interpersonal aspect) in this context is understood as compliance, kindness, uncritical adjustment to others, dependence, inability to refuse, sacrificing oneself for others. Restricted affectivity (the interpersonal aspect) manifests itself in the repression and suppression of negative emotions, low awareness of experienced emotions, passiveness, and helplessness.

Research exists which supports personality as a risk factor for cancer (5–9). However, the results of well-conducted prospective studies and metanalyses have not confirmed an association between personality traits and cancer risk (10–13). Due to its superior sample size and its methodological strength, the Nakaya study, which investigated neuroticism and extraversion as possible risk factors for cancer, might for many researchers be the final proof that such a connection does not exist (11). According to Ranchor et al. (14) the overall effect size for a personality-cancer causal association is not sufficient to assume there may in fact be clinical and public health consequences. The question which still begs to be posed is whether, due to a lack of evidence from large prospective studies which would confirm such a cause-effect association, it is legitimate to deny its existence.

The senior author's clinical experience of over 20 years of working with patients with emotional disorders indicates that on a level of particular patients such a connection might indeed exist. There have been two clinical cases which have especially stuck with the author, i.e., two patients both of whom were diagnosed with cancer in 2020–a 58-year-old woman who got breast cancer and a 44-year-old man who got melanoma malignum (the patient died on January 4^th^, 2021). What links these two cases is that for several years both of these patients had been undergoing pharmacological treatment for depressive disorders and were referred to a psychotherapist by the author. Psychotherapy was suggested due to the patients' poor anger management skills which led them to deny and block it so much so they were consciously not experiencing it when their borders had been breached. During one of the author's conversations with their therapist on the patient's condition she spontaneously remarked that should they go on like this, they would soon get cancer. The statement was made 2 years prior to the patient's diagnosis; the question which arises then is why something which in interpersonal contact the therapist found obvious is not detected on the level of prospective studies.

As the authors see it, the reason may be that prospective studies account only for selected aspects of personality such as for example personality traits of the Five Factor Model (neuroticism, extraversion) without taking stock of non-specific, non-homogeneous or incommensurable factors such as each patient's unique life story, chance events and resultant stress, coping methods or the impact of social support. It would indeed be very difficult to include patient's unique life stories and their social relations in prospective studies; therefore, the authors cannot stress enough the importance of conducting case-control studies because results thus obtained might help improve designing prospective studies.

The distressed personality (Type D personality) concept was developed in the 1990's by Johan Denolett. Denolett suggested it may be a significant risk factor in cardiovascular diseases, in particular hypertension and ischemic heart disease (15). Type D is composed of two personality traits: Negative Affectivity (NA) and Social Inhibition (SI) (16). High score on the NA scale indicates the tendency to experience negative emotions, whereas high score on the SI spectrum means inhibition of emotional expression and behavior. Patients with established coronary artery disease are at greater risk of a heart attack if they have Type D personality (17).

There are few studies of Type D personality in the field of oncology. The existing studies mainly focus on colorectal cancer survivors (CRC) among whom nineteen percent can be classified as having a Type D personality (18). These patients have a higher number of comorbidities and an increased health care utilization (19). CRC patients with elevated levels of NA have an increased risk of all-cause mortality (20).

The goal of this study is to establish the differences in Type D personality and the Big Five Personality traits between a group of newly diagnosed breast cancer patients and a group of controls.

## Methods

### Study Design and Participants

A comparative study of breast cancer patients and women without previous history of cancer was carried out. Inclusion criteria entailed age between 18 and 80, diagnosis of breast cancer (C50 according of ICD-10), no history of any serious life-threatening diseases or any serious psychiatric disorders (Major depression, Schizophrenia, Bipolar disorders).

Subjects in the breast cancer group were consecutively referred to the Breast Unit at the Lower Silesian Oncology Centre in Wroclaw, for active surgical treatment carried out between December 2015 and January 2017. The average time from diagnosis of breast cancer to admission to the Breast Unit for surgery was 2 months.

Women in the “comparison group” were recruited from the general population.

Prior to data collection, study approval was obtained from the local ethics committee (KB- 619/2015) and performed in accordance with the Helsinki ethical standard.

### Measures

Medical and sociodemographic data were obtained from the participants and from their medical files, and recorded using a self-evaluation questionnaire. In order to measure personality traits and depressive symptoms the following measuring instruments were applied:

The NEO Personality Inventory (NEO-FFI)–a self-administered questionnaire, consisting of 60 statements that are rated on a 5-point Likert scale, ranging from 1- “completely disagree” to 5- “completely agree” (5). The inventory is based on the theory of the Big Five personality traits and allows to assess personality dimensions such as openness to experience, conscientiousness, extraversion, agreeableness, neuroticism (21). The scores of all items were summed up to provide an overall measure of each personality trait. Raw scores were normalized for the Polish population through an interpretation based on a ten-item sten scale. Sten norms were determined for 5 age groups (15–19, 20–29, 30–39, 40–49, 50–80), separately for men and women. Result interpretation on the basis of sten points indicates the following: 1–3 low trait intensity; 4–6 average; 7–10 high trait intensity. The measurement has been translated and validated to use in the Polish population (Cronbach's alpha ranged from 0.68 to 0.82).

Type D Scale-14 is a well-established and widely used instrument for the assessment of the type-D personality pattern (16). The scale is a self-administered instrument that consists of 14 items comprising two subscales: a 7-item subscale that measures Negative Affectivity and a 7-item subscale measuring Social Inhibition.

A patient conducts a self-assessment of their own behavior using a 5-item scale in which 0 means a false statement, and 4 a true statement. The NA and SI scales can be scored as continuous variables (range 0–28) to assess these personality traits independently. A person manifests a type-D personality if his/her result on both scales (SI and NA) equals or exceeds 10.

The measurement has been translated and validated to use in the Polish population with satisfactory internal consistency (Cronbach's alpha > 0.80).

### Statistical Analysis

The Shapiro-Wilk test and visual assessment of distribution were used to analyze the normality of the data. Comparisons between the female breast cancer and no cancer control groups were carried out using the Chi-squared test or Fisher's exact test for categorical variables, and Mann–Whitney *U* test for quantitative variables. Multivariate logistic regression was used to assess independent effect personality traits and how they correlate with belonging to the breast cancer group. To exclude confounding effects, all models were adjusted for age. The no cancer control group was used as the reference group and 95% CI were calculated for all odds ratios.

Significance level was set to *p* < 0.05. Analyses were performed using statistical software package R for Windows (version 4.0.3, R Foundation for Statistical Computing, Vienna, Austria).

## Results

### Subjects Characteristics

Among the 200 women with breast cancer contacted, 105 accepted to participate (52.5%). The stated reasons for refusal included “lack of interest,” “being too tired,” and “not wishing to speak about illness.” Among 105 women who accepted to participate, 99 fulfilled the inclusion criteria. The 6 non-eligible women were excluded due to their diagnosis being Benign neoplasm of breast (ICD-10 D24).

Of the 100 participants in the comparison group recruited in the study, only women with no missing data for all the variables considered in the analysis were included.

The present analysis was thus conducted on 178 subjects (99 with breast cancer and 79 non-exposed). The cancer and comparison groups were found to differ with regard to age (*p* = 0.000), but not marital status (*p* = 0.303), education level (*p* = 0.126) or having children (*p* = 0.278) (Table 1).

**Table 1:** Sociodemographic characteristic of participants.

	**Breast cancer** **group n=99**		**Comparison** **group n=79**		***p*-Value**
Mean age (SD)	58.9 (10.3)		47.7 (12.6)		0.000
Marital status					0.303
Single divorced/separated)	34	34.34 %	20	25.98 %	
Married/cohabiting	65	65.66 %	57	74.02 %	
Education (years)					0.126
≤ 8	5	5.05 %	2	2.53 %	
8–10	13	13.13 %	6	7.%	
11–12	53	53.54 %	36	45.57 %	
≥12	28	28.28 %	35	44.30 %	
Children					0.278
No	11	11.11 %	15	18.99 %	
Yes	88	88.89 %	64	81.01 %	

### NEO-FFI

Analysis of personality traits measured in NEO-FFI revealed that in the breast cancer group the percentage of participants with Neuroticism HIGH (25.25 %) is statistically significantly larger than in the control group (10.12%) (Table 2).

**Table 2:** Scores on the big five personality model for the breast cancer and control groups.

**NEO-FFI Dimension**	**Sten scores**			**Sten scores categories LOW (stens 1-3) MID (stens 4-6) HIGH (stens 7-10)**						
	**Breast cancer group *n* = 99**	**Comparison group *n* = 79**	***p*-value**	**Breast cancer group** ***n*** **=** **99**			**Comparison group** ***n*** **=** **79**			***p*-value and *post-hoc* contrast**
	**M ±SD**	**M ±SD**		**LOW**	**MID**	**HIGH**	**LOW**	**MID**	**HIGH**	
Neuroticism	4.72 ± 2.04	4.24 ± 1.77	0.09	26.26%	48.48%	25.25%	36.70%	53.16%	10.12%	**0.02 L vs. H 0.04**
Extraversion	5.63 ± 1.99	6.11 ± 1.06	0.14	17.17%	46.46%	36.36%	5.06%	50.63%	44.30%	0.08
Openness to experience	5.61 ± 2.17	5.30 ± 1.98	0.39	19.19%	50.50%	30.30%	20.25%	53.16%	26.58%	0.86
Agreeableness	6.09 ± 1.80	5.74 ± 1.79	0.17	11.11%	45.45%	43.43%	8.86%	59.49%	31.64%	0.17
Conscientiousness	5.72 ± 1.59	5.75 ± 1.79	0.98	9.09%	54.54%	36.36%	8.86%	53.16%	37.97%	0.97

*Bold values means statistically significant*.

### DS-14

Among breast cancer patients, 40 (40.40%) had type D personality as compared to 22 (27.84%) of the controls. However, the difference between the groups was not statistically significant (*p* = 0.11).

67 % Breast Cancer Patients Exhibited Negative Affectivity in Comparison to 35 % in Control Group (*p* = 0.000). 51 % of Patients Had Social Inhibition in Comparison to 46 % of Controls (NS). The Mean Score in Negative Affectivity Scales Was Significantly Higher in the Breast Cancer Group-12.65 (6.19 SD) Compared to 8.12 (5.06 SD) in Controls (*p* = 0.000).

The mean score in Social Inhibition scales was statistically not particularly different between the group of patients (10.41 ± 6.32) and the group of controls (9.02 ± 6.38).

### Multivariate Logistic Regression

Multivariate logistic regression revealed the following (Table 3). Participants with Neuroticism High and Openness to experience HIGH had a higher risk of belonging to the breast cancer group (OR 3.97 and OR 3.47). From the analyzed personality traits, the highest risk (OR 4.45) of belonging to the group of patients was linked to Negative Affectivity while Social Inhibition decreased that risk (OR 0.40) (Table 3).

**Table 3:** Multivariate logistic regression model predicting belonging to breast cancer group based on personality traits.

**Risk factor**	**OR**	**95% CI**	*p*-value^*^
Neuroticism MID	1.43	0.59–3.48	0.41
Neuroticism HIGH	3.97	1.08–15.81	**0.04**
Openness to experience MID	2.11	0.78–5.91	0.14
Openness to experience HIGH	3.47	1.11–11.49	**0.03**
Negative affectivity	4.45	1.96–10.61	**0.000**
Social inhibition	0.40	0.16–0.92	**0.03**
Age	1.09	1.06–1.13	**0.000**

* *p <0.005. Bold values means statistically significant*.

## Discussion

The main finding of our study is that Negative Affectivity is associated factor (OR 4.45) to the risk of the breast cancer group, while SI lowers the risk (OR 0.40). The precedence of type D personality, referring to the combined traits of NA and SI, does not differ significantly between the groups.

The obtained results indicate that the tendency to experience increased negative emotions (NA) was linked to belonging to the breast cancer group, whereas inhibition of emotional and behavioral expression (SI) decreased this risk. The outcome surprised scholars because cancer-personality is associated with neuroticism and introversion (1, 2). De Fruyt and Denollet (22) found half of the variance of both SI and NA to be predicted by five-factor model dimensions, with SI showing strong negative associations with extraversion (*r* = −0.61) and NA strong positive associations with neuroticism (*r* = +0.74). In our investigation neuroticism was second to NA among factors increasing the risk of belonging to the breast cancer group (OR 3.97). Extraversion, in turn, was not such a factor. Denollet suggested that the SI trait is not identical to the introversion trait. The SI trait focuses on the interpersonal dimension of introversion (low self-expression) rather than the intrapsychic (positive affect, excitement seeking) dimension (23). The results of Howard's study also suggest that although NA and neuroticism can be considered synonymous, the relationship between SI and Introversion is not as clear-cut as it may have been supposed, with the SI trait differing from introversion (24).

The findings of our study according to which the tendency to experience increased negative emotions (NA) is associated factor to the risk for belonging to the breast cancer group, whereas inhibition of emotional and behavioral expression (SI) lowers that risk contribute to the discussion on emotion regulation mechanisms and their role in cancer. Regulating emotional distress by active rather than evasive strategies is considered more adaptive because it relates to lower depression (25). Women who reported coping with cancer by expressing emotions reported fewer depressive symptoms 3 months later as compared to those with low emotional expression (26). According to Aldao et Nolen-Hoeksema, acceptance, reappraisal, problem solving as adaptive emotion regulation strategies show weaker associations with psychopathology than suppression of experience, worry avoidance, or rumination, which are maladaptive strategies (27). Referring to research on coping through emotional approach Stanton at al. distinguishes two major factors–emotional processing (active attempts to acknowledge and understand emotions) and emotional expression (28). This particular finding supports the notion that expressing emotions actively rather than avoiding them is an adaptive response to the challenges of emotional distress which stands in opposition to our results according to which inhibition of emotional and behavioral expression (SI) lowered the risk of belonging to the breast cancer group. Our findings, though, corroborate the research outcomes obtained by Lemogne et al. (7). In this prospective cohort study Type 1 personality, characterized by suppressed emotional expression in interpersonal relationships, was associated with a decreased risk of breast cancer (7). How, then, to account for these contradictory findings? Detailed analysis reveals that in fact this may only be an apparent contradiction. In their study of the relationships between rumination and other coping or emotion-regulation strategies (29), Nolen-Hoeksema et al. note that attempts to regulate negative emotions in the form of repetitive (i.e., rumination) processing give rise to maladaptive outcomes, including depression and anxiety. Rumination on negative affect can begin as an active coping effort but persists as passive, perseverative dwelling on distress (30). Therefore, it transpires that not all forms of focus on one's emotions are adaptive. To the question of what determines whether emotional expression and processing are linked with positive vs. negative mental health outcomes Marroquin replies that it partly depends on intrapersonal factors and environmental contexts (31). In his research he has proven that women whose written narratives of cancer experience included higher proportions of words for negative emotions reported more depressive symptoms 3 months later. Thus, implicit expression of negative emotion during essay-writing predicted more depressive symptoms. Additionally, he revealed that implicit loneliness moderates the effects of emotional approach.

The authors believe that the results they obtained point toward aspects related to the significance of emotion expression as a healthy mechanism of its regulation, i.e., a factor which decide about the ambiguity of studies on the association between personality and cancer. First, the author's psychotherapeutic experience indicated that expressing emotions is not always identical with their conscious experiencing–and it is the high awareness of experienced emotions, the ability to identify and name them which facilitates the organism's auto-regulation. Second, awareness of experienced emotions at the moment of a particular emotion's occurrence in the organism plays an important role in fostering a healthy auto-regulation because it enables expressing emotions for the purpose of neutralizing the factor which engendered them, e.g., expressing anger in order to protect our boundaries when they are being breached. As the authors see it, expressing anger post factum–without being aware of its causes–might only lead to estranging others and result in loneliness and lack of social support, especially when the person does not understand the reasons of the situation and hence does not take responsibility for it. Therefore, paradoxically in those cases when a person is not aware of their negative emotions (NA, neuroticism)–and is not able to relate them to factors which might be causing them–SI might perform a protective function not allowing for their external expression which could only deepen a feeling of loneliness. All these aspects are easily readable in the patient-therapist relationship but prove difficult or even impossible to capture in prospective studies.

The second finding of our study is that for participants with high neuroticism (7-10 stens–Neurotism HIGH) the odds ratio for entering the breast cancer group was 3.97. These results correspond with research indicating that neuroticism and negative affect are related to cancer onset and mortality (5, 6, 8, 9, 32).

Higher neuroticism is associated with difficulty in regulating negative emotions (33).

Those high on the neuroticism scale manifest heightened degrees of anxiety, vulnerability, sadness, angry hostility, irritability, impulsiveness, fearfulness, helplessness, self-consciousness, embarrassment, and the inability to cope with stress.

High neuroticism is associated primarily with negative health behaviors (34, 35). Otonari et al. (9) found in a prospective study that women with a higher neuroticism trait had a higher cancer risk, and neuroticism was positively associated with risk factors for cancer (women with higher neuroticism consumed less fruit and vegetables, performed less exercise, had a higher smoking rate and higher risk BMI values).

Neuroticism might affect the immune system by changing the quality and quantity of stressors, leading not only to a greater presence of stressors (i.e., negative health behaviors) but also influencing the response to stressors (36). In more neurotic individuals higher levels of physiological biomarkers of chronic inflammation such as interleukin-6 and C-reactive protein (37), as well as peripheral blood mononuclear cells have been found (38, 39).

The third finding of our study posits that individuals with high results on the openness to experience scale had a higher associated factor to the risk of entering the breast cancer group. Openness reflects an open attitude toward experiences, beliefs and people. Individuals with high results on the openness scale are curious and unconventional. Nolan et al. found openness to experience as a significant predictor of breast lump check and mammogram while Bahat found that High extraversion, high openness and high conscientiousness predicted a greater participation in breast self-examination (40, 41). Study of Iwasa et al. (42) revealed that older individuals high in openness to experience are likely to take part in checkups for the elderly.

Boeft et al. revealed an association between increased openness to experience and an increased number of medical services and Toivonen et al. (43, 44) found greater openness to experience related to use of Complementary Therapy use after breast cancer treatment.

Our finding that individuals with high results on the openness to experience scale had a higher associated factor to the risk of entering the breast cancer group according to the authors, could be connected with the treatment Centre in which our study was carried out.

Breast cancer is the most common cancer among Polish women. Each year over 18 000 women hear such a diagnosis and one in three cases end in the patient's death because the breast cancer is still very frequently diagnosed in an advanced stage. The reason for this is that women are afraid of oncological treatment, incl. due to the many negative changes in body image during treatment (45). The Lower Silesian Oncology Centre being the largest oncological treatment center in the region, causes fear in most people entering it. Patients and their family members admit it when talking to staff. Breast Unit at the Lower Silesian Oncology Centre is a reference center for the treatment of breast cancer and one of the best centers in Poland. However, despite this some patients with breast cancer often admit in conversations that they prefer to choose oncology departments in general hospitals as the place of treatment, because it is associated with less anxiety for them. So, this could explain the fact that the Breast Unit at the Lower Silesian could be predominantly chosen by women with high results on the openness scale which reflects intellectual stimulation, enjoyment of novelty, change and variety.

### Study Limitations

The study was carried out on a small population sample. Personality traits were assessed for 52% of patients who reported for treatment and gave their consent for participating in the study, which might mean it is unrepresentative. Since different geographic populations have different dispositions, this study surveyed populations only from Poland. Given the cross-sectional design of the investigation, prospective studies are needed to understand the causal nature of our results.

### Clinical Implications

Neuroticism and negative affectivity were the strongest associated factors to the risk of the breast cancer group in comparison to the control group. Patients with such a personality profile in groups of increased breast cancer risk should be especially targeted for psychological and social support.

## Conclusions

This was the first case-control study which analyzed the NA and SI traits in breast cancer patients. NA was significantly associated factor related to an increased risk for belonging to breast cancer, whereas, SI was associated with a decreased risk of breast cancer. Inhibition of emotional expression (SI) as an associated factor of decreased risk of breast cancer might indicate that expressing negative emotions is not always a healthy mechanism of their regulation. The authors believe that these findings might contribute to a better understanding of the association between personality traits and breast cancer.

## Data Availability Statement

The raw data supporting the conclusions of this article will be made available by the authors, without undue reservation.

## Ethics Statement

The studies involving human participants were reviewed and approved by Wroclaw Medical Univeristy Ethics Committee. The patients/participants provided their written informed consent to participate in this study.

## Author Contributions

TP, IW, and RM contributed to conception, design of the study, and wrote sections of the manuscript. IW organized the database and performed the statistical analysis. IW and TP wrote the first draft of the manuscript. All authors contributed to manuscript revision, read, and approved the submitted version.

## Funding

This study was supported by Wroclaw University Grant STM.C230.16.024 and SUB.C230.21.013.

## Conflict of Interest

The authors declare that the research was conducted in the absence of any commercial or financial relationships that could be construed as a potential conflict of interest.

## Publisher's Note

All claims expressed in this article are solely those of the authors and do not necessarily represent those of their affiliated organizations, or those of the publisher, the editors and the reviewers. Any product that may be evaluated in this article, or claim that may be made by its manufacturer, is not guaranteed or endorsed by the publisher.
